# 3-(*m*-Tol­yloxy)phthalonitrile

**DOI:** 10.1107/S1600536807068225

**Published:** 2008-01-09

**Authors:** Xian-Fu Zhang, Dandan Jia, Qiang Liu, Aijun Song

**Affiliations:** a Department of Chemistry, Hebei Normal University of Science and Technology, Qinhuangdao, Hebei Province 066004, People’s Republic of China; b Department of Chemistry, Beijing University of Chemical Technology, Beijing 100029, People’s Republic of China

## Abstract

In the mol­ecule of the title compound, C_15_H_10_N_2_O, the dihedral angle between the two benzene rings is 65.49 (9)°.

## Related literature

For the synthesis of a related compound, see: Sharman & van Lier (2003 ▶). For the crystal structure of an isomer of the title compound see: Ocak Ískeleli (2007 ▶). For related literature, see: Atalay *et al.* (2003 ▶, 2004 ▶); Cave *et al.* (1986 ▶); Koysal *et al.* (2004 ▶); Leznoff & Lever (1989–1996 ▶); McKeown (1998 ▶); Ocak *et al.* (2003 ▶).
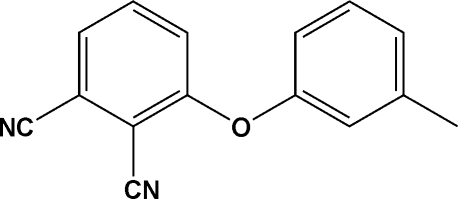


## Experimental

### 

#### Crystal data

C_15_H_10_N_2_O
*M* *_r_* = 234.25
Orthorhombic, 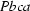

*a* = 25.514 (3) Å
*b* = 14.6064 (18) Å
*c* = 6.6109 (6) Å
*V* = 2463.7 (5) Å^3^
*Z* = 8
Mo *K*α radiation
μ = 0.08 mm^−1^
*T* = 295 (2) K
0.6 × 0.5 × 0.3 mm

#### Data collection

Bruker P4 diffractometer
Absorption correction: none
3094 measured reflections
2254 independent reflections
1372 reflections with *I* > 2σ(*I*)
*R*_int_ = 0.041
3 standard reflections every 97 reflections intensity decay: none

#### Refinement

*R*[*F* ^2^ > 2σ(*F* ^2^)] = 0.054
*wR*(*F* ^2^) = 0.109
*S* = 1.04
2254 reflections
165 parameters
H-atom parameters constrained
Δρ_max_ = 0.21 e Å^−3^
Δρ_min_ = −0.23 e Å^−3^

### 

Data collection: *XSCANS* (Bruker, 1997 ▶); cell refinement: *XSCANS*; data reduction: *XSCANS*; program(s) used to solve structure: *SHELXTL* (Bruker, 1997 ▶); program(s) used to refine structure: *SHELXTL*; molecular graphics: *SHELXTL*; software used to prepare material for publication: *SHELXTL*.

## Supplementary Material

Crystal structure: contains datablocks I, global. DOI: 10.1107/S1600536807068225/rk2070sup1.cif

Structure factors: contains datablocks I. DOI: 10.1107/S1600536807068225/rk2070Isup2.hkl

Additional supplementary materials:  crystallographic information; 3D view; checkCIF report

## Figures and Tables

**Fig. 1.**
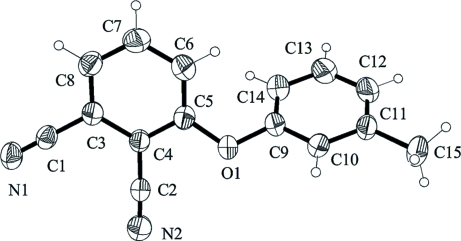
The molecular structure of title compound with the atom numbering scheme. Displacement ellipsoids are drawn at 35% probability level. Hydrogen atoms are presented as spheres of arbitrary radius.

**Fig. 2.**
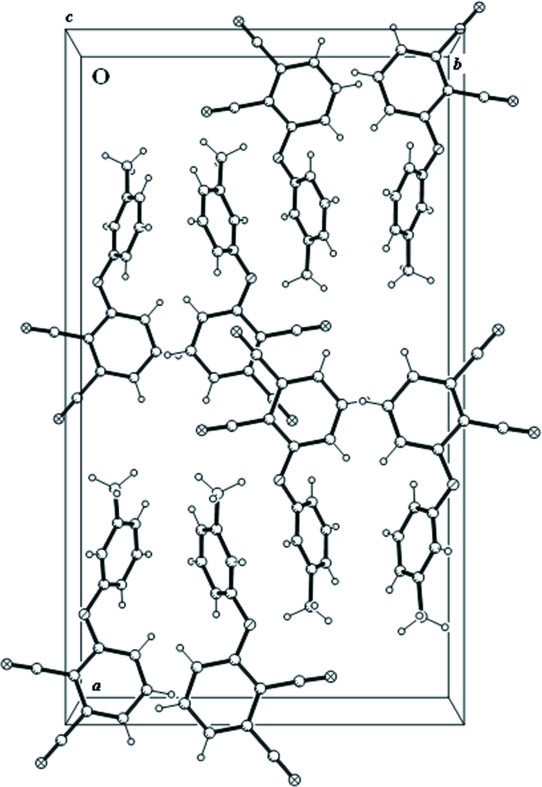
The packing of (I), viewed down the c–axis.

## supplementary crystallographic information

### Comment

Phthalonitriles are among the most important precusors of phthalocyanine materials (Leznoff, 1989–1986). Mono phenyloxyphthalonitriles have been used for preparing symetrical phthalocyanines and subphthalocyanines which have been applied in many areas, such as laser printing, photocopying, optical data storage, catalyst *etc*. (McKeown, 1998). The 3–(*m*–tolyloxy)phthalonitrile (I), which contains an electron–donating moiety and a strong electron–accepting fragment linked by an oxygen atom, is also a good model suitable for the study of photoinduced electron transfer between the short linked donor and acceptor. The rate of such electron transfer process and the lifetime of the resultant charge separation state, however, are highly dependent on the relative orientation between the donor and the acceptor (Cave, 1986). The crystal structure of the title compound, (I), can therefore provide very helpful information for it.

The triple bond lengths between C and N, both 1.140 (3)Å and 1.133 (3) Å, as shown in Fig. 1, agree with literature values (Ocak *et al.*, 2003). The geometry around the O atoms is in good agreement with the literature (Atalay *et al.*, 2003, 2004; Koysal *et al.*, 2004). The dihedral angle between the two aromatic rings planes is 65.49 (9)°. The crystal structure of compound involves extensive intermolecular π–π interactions, as can be seen from the packing diagram (Fig. 2). Phthalonitrile moieties are packed shoulder by shoulder along the *a*–axis which is stablized by the intermolecular dipole–dipole interactions and partial face–to–face π–π overlaping along the *c*–axis, while the toluene moieties are arranged by face to face π–π stacking along the *b*–axis and shouler by shoulder along the *c*–axis within the distance 4.15–4.20 Å. It is worth noting that the structure of the isomeric 4–(*m*–tolyloxy)phthalonitrile is monoclinic (Ocak Ískeleli, 2007) while the title compound report herein is orthorhombic.

### Experimental

The *m*–cresol (1.56 g, 14.4 mmol) and 3–nitrophthalonitrile (1.60 g, 9.3 mmol) were dissolved in dry DMF (30 ml) with stirring under N_2_. Dry fine–powdered potassium carbonate (2.5 g, 18.1 mmol) was added in portions evenly every 10 min. The reaction mixture was stirred for 48 h at room temperature and poured into iced water (150 g). The product was filtered off and washed with (10% *w*/*w*) NaOH solution and water until the filtrate was neutral. Recrystallization from ethanol gave a white product (yield 1.2 g, 55%). Single crystals were obtained from absolute ethanol at room temperature *via* slow evaporation (m.p. 374–375 K). IR data (ν_max_/cm^-1^): 3086 (*Ar*—H), 2980–2950 (CH_3_), 2229 (CN). ^1^H NMR data (p.p.m.): 2.34 (s,3*H*), 7.00–7.05 (d, 1H), 7.07 (s, 1H), 7.12–7.17 (d, 1H), 7.24–7.29 (dd, 1H), 7.35–7.42 (t, 1H), 7.81–7.84 (d, 1H), 7.84–7.85 (d, 1H).

### Refinement

All H atoms were positioned geometrically and refined using a riding model, with C—H = 0.96 Å (CH_3_) and C—H = 0.93 Å (CH) with *U*_iso_(H) = 1.2*U*_eq_ (parent C) (for CH) or *U*_iso_(H) = 1.5*U*_eq_ (parent C) (for CH_3_).

### Crystal data

**Table:**

C_15_H_10_N_2_O	*D*_x_ = 1.263 Mg m^−^^3^
*M**_r_* = 234.25	Melting point: 374 K
Orthorhombic, *P**b**c**a*	Mo *K*α radiation λ = 0.71073 Å
Hall symbol: -P 2ac 2ab	Cell parameters from 58 reflections
*a* = 25.514 (3) Å	θ = 2.8–25.5º
*b* = 14.6064 (18) Å	µ = 0.08 mm^−^^1^
*c* = 6.6109 (6) Å	*T* = 295 (2) K
*V* = 2463.7 (5) Å^3^	Prism, colourless
*Z* = 8	0.6 × 0.5 × 0.3 mm
*F*_000_ = 976	

### Data collection

**Table:**

Bruker P4 diffractometer	*R*_int_ = 0.041
Radiation source: fine–focus sealed tube	θ_max_ = 25.5º
Monochromator: graphite	θ_min_ = 2.1º
*T* = 295(2) K	*h* = −30→1
ω scans	*k* = −17→1
Absorption correction: none	*l* = −1→8
3094 measured reflections	3 standard reflections
2254 independent reflections	every 97 reflections
1372 reflections with *I* > 2σ(*I*)	intensity decay: none

### Refinement

**Table:**

Refinement on *F*^2^	Hydrogen site location: inferred from neighbouring sites
Least-squares matrix: full	H-atom parameters constrained
*R*[*F*^2^ > 2σ(*F*^2^)] = 0.054	*w* = 1/[σ^2^(*F*_o_^2^) + (0.001*P*)^2^ + 2.2*P*] where *P* = (*F*_o_^2^ + 2*F*_c_^2^)/3
*wR*(*F*^2^) = 0.109	(Δ/σ)_max_ < 0.001
*S* = 1.04	Δρ_max_ = 0.21 e Å^−^^3^
2254 reflections	Δρ_min_ = −0.23 e Å^−^^3^
165 parameters	Extinction correction: SHELXTL (Bruker, 1997), Fc^*^=kFc[1+0.001xFc^2^λ^3^/sin(2θ)]^-1/4^
Primary atom site location: structure-invariant direct methods	Extinction coefficient: 0.00028 (6)
Secondary atom site location: difference Fourier map	

### Special details

**Table:**

Geometry. All s.u.'s (except the s.u.'s in the dihedral angle between two l.s. planes) are estimated using the full covariance matrix. The cell s.u.'s are taken into account individually in the estimation of s.u.'s in distances, angles and torsion angles; correlations between s.u.'s in cell parameters are only used when they are defined by crystal symmetry. An approximate (isotropic) treatment of cell s.u.'s is used for estimating s.u.'s involving l.s. planes.
Refinement. Refinement of *F*^2^ against ALL reflections. The weighted *R*–factor *wR* and goodness of fit *S* are based on *F*^2^, conventional *R*–factors *R* are based on *F*, with *F* set to zero for negative *F*^2^. The threshold expression of *F*^2^ > 2σ(*F*^2^) is used only for calculating *R*–factors(gt) *etc*. and is not relevant to the choice of reflections for refinement. *R*–factors based on *F*^2^ are statistically about twice as large as those based on *F*, and *R*–factors based on ALL data will be even larger.

### Fractional atomic coordinates and isotropic or equivalent isotropic displacement parameters (Å^2^)

**Table:**

	*x*	*y*	*z*	*U*_iso_*/*U*_eq_	
O1	0.65134 (6)	0.53717 (13)	0.7428 (3)	0.0755 (6)	
N1	0.43352 (8)	0.42711 (17)	0.7588 (4)	0.0682 (7)	
N2	0.57804 (9)	0.33811 (18)	0.7422 (5)	0.0842 (8)	
C1	0.46678 (9)	0.47844 (18)	0.7550 (4)	0.0532 (6)	
C2	0.57161 (9)	0.41537 (19)	0.7437 (4)	0.0570 (7)	
C3	0.50933 (9)	0.54397 (17)	0.7508 (4)	0.0510 (6)	
C4	0.56143 (9)	0.51093 (16)	0.7447 (4)	0.0499 (6)	
C5	0.60184 (9)	0.57428 (18)	0.7405 (4)	0.0568 (6)	
C6	0.59127 (11)	0.66675 (19)	0.7441 (5)	0.0678 (8)	
H6A	0.6187	0.7086	0.7429	0.081*	
C7	0.54050 (11)	0.69742 (19)	0.7495 (5)	0.0708 (8)	
H7A	0.5338	0.7600	0.7514	0.085*	
C8	0.49907 (11)	0.63599 (19)	0.7523 (4)	0.0634 (7)	
H8A	0.4647	0.6571	0.7551	0.076*	
C9	0.69296 (10)	0.58418 (18)	0.6486 (5)	0.0641 (8)	
C10	0.74003 (9)	0.58177 (18)	0.7517 (5)	0.0647 (7)	
H10A	0.7423	0.5552	0.8792	0.078*	
C11	0.78408 (10)	0.62010 (19)	0.6599 (6)	0.0757 (9)	
C12	0.77921 (11)	0.6595 (2)	0.4711 (6)	0.0835 (10)	
H12A	0.8085	0.6851	0.4096	0.100*	
C13	0.73158 (12)	0.6617 (2)	0.3718 (6)	0.0822 (10)	
H13A	0.7289	0.6891	0.2452	0.099*	
C14	0.68779 (11)	0.6230 (2)	0.4610 (5)	0.0725 (8)	
H14A	0.6556	0.6233	0.3951	0.087*	
C15	0.83646 (11)	0.6162 (2)	0.7674 (7)	0.1120 (16)	
H15A	0.8590	0.6632	0.7152	0.168*	
H15B	0.8523	0.5574	0.7455	0.168*	
H15C	0.8313	0.6255	0.9098	0.168*	

### Atomic displacement parameters (Å^2^)

**Table:**

	*U*^11^	*U*^22^	*U*^33^	*U*^12^	*U*^13^	*U*^23^
O1	0.0412 (9)	0.0688 (12)	0.1166 (18)	−0.0042 (8)	0.0010 (11)	0.0251 (12)
N1	0.0486 (12)	0.0869 (17)	0.0691 (16)	−0.0053 (12)	−0.0033 (12)	0.0028 (14)
N2	0.0641 (15)	0.0659 (16)	0.123 (3)	0.0005 (12)	0.0043 (16)	0.0054 (17)
C1	0.0453 (13)	0.0675 (16)	0.0469 (14)	0.0045 (12)	−0.0021 (12)	0.0014 (13)
C2	0.0411 (12)	0.0611 (16)	0.0688 (18)	−0.0028 (12)	0.0024 (13)	0.0046 (15)
C3	0.0461 (12)	0.0618 (15)	0.0452 (14)	0.0005 (11)	−0.0002 (12)	0.0005 (12)
C4	0.0433 (12)	0.0564 (14)	0.0500 (14)	0.0003 (11)	0.0015 (12)	0.0024 (13)
C5	0.0447 (12)	0.0627 (15)	0.0630 (17)	−0.0007 (11)	−0.0008 (13)	0.0029 (14)
C6	0.0603 (16)	0.0609 (16)	0.082 (2)	−0.0092 (13)	0.0012 (16)	0.0005 (16)
C7	0.0757 (18)	0.0554 (15)	0.081 (2)	0.0070 (14)	0.0064 (17)	−0.0022 (16)
C8	0.0555 (14)	0.0682 (17)	0.0665 (17)	0.0106 (13)	0.0057 (14)	0.0006 (15)
C9	0.0456 (13)	0.0553 (15)	0.091 (2)	−0.0074 (12)	0.0025 (15)	0.0055 (16)
C10	0.0458 (13)	0.0570 (15)	0.091 (2)	0.0012 (12)	−0.0044 (15)	0.0047 (16)
C11	0.0420 (14)	0.0559 (16)	0.129 (3)	0.0002 (12)	−0.0001 (17)	−0.0025 (19)
C12	0.0565 (17)	0.0668 (19)	0.127 (3)	−0.0029 (14)	0.0243 (19)	0.012 (2)
C13	0.075 (2)	0.075 (2)	0.097 (3)	−0.0020 (16)	0.0121 (19)	0.0110 (19)
C14	0.0572 (16)	0.0723 (19)	0.088 (2)	−0.0070 (14)	−0.0036 (16)	0.0037 (18)
C15	0.0446 (15)	0.090 (2)	0.202 (5)	−0.0048 (15)	−0.022 (2)	0.013 (3)

### Geometric parameters (Å, °)

**Table:**

O1—C5	1.375 (3)	C9—C14	1.370 (4)
O1—C9	1.409 (3)	C9—C10	1.381 (4)
N1—C1	1.133 (3)	C10—C11	1.394 (4)
N2—C2	1.140 (3)	C10—H10A	0.9300
C1—C3	1.448 (3)	C11—C12	1.380 (5)
C2—C4	1.420 (4)	C11—C15	1.515 (4)
C3—C8	1.369 (4)	C12—C13	1.382 (4)
C3—C4	1.415 (3)	C12—H12A	0.9300
C4—C5	1.386 (3)	C13—C14	1.384 (4)
C5—C6	1.377 (4)	C13—H13A	0.9300
C6—C7	1.371 (4)	C14—H14A	0.9300
C6—H6A	0.9300	C15—H15A	0.9600
C7—C8	1.387 (4)	C15—H15B	0.9600
C7—H7A	0.9300	C15—H15C	0.9600
C8—H8A	0.9300		
			
C5—O1—C9	119.7 (2)	C10—C9—O1	115.2 (3)
N1—C1—C3	179.8 (3)	C9—C10—C11	118.4 (3)
N2—C2—C4	177.7 (3)	C9—C10—H10A	120.8
C8—C3—C4	121.0 (2)	C11—C10—H10A	120.8
C8—C3—C1	120.4 (2)	C12—C11—C10	119.2 (3)
C4—C3—C1	118.7 (2)	C12—C11—C15	121.3 (3)
C5—C4—C3	118.2 (2)	C10—C11—C15	119.5 (3)
C5—C4—C2	121.3 (2)	C11—C12—C13	121.2 (3)
C3—C4—C2	120.5 (2)	C11—C12—H12A	119.4
O1—C5—C6	124.5 (2)	C13—C12—H12A	119.4
O1—C5—C4	114.8 (2)	C12—C13—C14	119.9 (3)
C6—C5—C4	120.6 (2)	C12—C13—H13A	120.1
C7—C6—C5	120.4 (2)	C14—C13—H13A	120.1
C7—C6—H6A	119.8	C9—C14—C13	118.5 (3)
C5—C6—H6A	119.8	C9—C14—H14A	120.8
C6—C7—C8	120.6 (3)	C13—C14—H14A	120.8
C6—C7—H7A	119.7	C11—C15—H15A	109.5
C8—C7—H7A	119.7	C11—C15—H15B	109.5
C3—C8—C7	119.3 (2)	H15A—C15—H15B	109.5
C3—C8—H8A	120.4	C11—C15—H15C	109.5
C7—C8—H8A	120.4	H15A—C15—H15C	109.5
C14—C9—C10	122.7 (3)	H15B—C15—H15C	109.5
C14—C9—O1	121.9 (3)		
